# Ultrasound-Assisted Extraction of Bioactive Compounds from Pomegranate Peel Using Vinegar with α-Cyclodextrin as a Green Solvent

**DOI:** 10.3390/foods15142446

**Published:** 2026-07-09

**Authors:** María de los Ángeles Martínez-Sánchez, Ginés Benito Martínez-Hernández, Antonio López-Gómez

**Affiliations:** 1 Food Safety and Refrigeration Engineering Group, Department of Agricultural Engineering, Universidad Politécnica de Cartagena, Paseo Alfonso XIII, 48, 30203 Cartagena, Spain; mariadelosangeles.martinez@edu.upct.es (M.d.l.Á.M.-S.); ginesbenito.martinez@upct.es (G.B.M.-H.); 2 Institute of Plant Biotechnology, Universidad Politécnica de Cartagena, Campus Muralla del Mar, Edificio I+D+I, 30202 Cartagena, Spain

**Keywords:** polyphenols, vinegar-based solvents, green extraction, punicalagin, DPPH radical scavenging activity, antimicrobial activity, agri-food byproducts

## Abstract

The recovery of polyphenols from pomegranate peel is limited by conventional extraction methods that rely on organic solvents. This study evaluated the ultrasound-assisted extraction (UAE) of dehydrated pomegranate peel using greener solvent systems based on distilled vinegar combined with α-cyclodextrin (αCD) to enhance the solubilization and recovery of phenolic compounds. The resulting extracts were characterized by total antioxidant capacity (DPPH free radical scavenging assay), total phenolic content (TPC, Folin–Ciocalteu assay), targeted phenolic analysis (HPLC-QTOF-MS analysis) and antimicrobial activity (Kirby–Bauer well-diffusion assay) against *Listeria monocytogenes* and *Salmonella enterica*. The unbuffered vinegar system (V2) exhibited the highest antioxidant capacity (2372.1 ± 109.7 µmol TE/g DW), significantly exceeding the methanolic control (1147.5 ± 477.6 µmol TE/g DW; *p* < 0.05). The vinegar-αCD system (V2-10) showed the highest TPC (196.2 ± 15.6 mg GAE/g DW), while HPLC-QTOF-MS identified punicalagin as the predominant phenolic compound (26.1–64.5 mg/g DW), with the highest total quantified phenolic content (76.2 mg/g DW). Vinegar-based extracts also inhibited *L. monocytogenes* (33.1–46.7 mm) and *S. enterica* (19.9–24.3 mm). Overall, UAE combined with vinegar-αCD solvents represents a promising green extraction strategy for obtaining polyphenol-rich extracts from pomegranate byproducts, providing comparative insights into solvent-dependent differences in phenolic profiles and bioactivity.

## 1. Introduction

Over the last few years, there has been increasing interest in the use of natural ingredients for food preservation, driven by the demand for healthier and more sustainable food systems [1,2]. Among these compounds, polyphenols have attracted significant attention due to their antioxidant and antimicrobial properties, as well as their potential application as natural preservatives and functional food ingredients [1,3,4,5].

At the same time, a humongous amount of fruit and vegetable byproducts is generated by the agri-food industry, representing a major environmental and economic challenge [6,7]. In this context, promoting circular economy and sustainability strategies such as valorization of these byproducts has emerged as a promising strategy, as they constitute a natural source of bioactive compounds like polyphenols [8,9,10]. Pomegranate peel is, in particular, known to be one of the most relevant plant byproducts due to its high content of phenolic compounds such as ellagitannins [11,12,13].

The predominant phenolic compounds identified in pomegranate peel include punicalagins (α- and β-isomers), ellagic acid, gallic acid, and other ellagitannin derivatives, with punicalagins being the major contributors to its exceptionally high antioxidant capacity. In addition to their antioxidant activity, these compounds have demonstrated antimicrobial and anti-inflammatory properties, making pomegranate peel extracts attractive candidates for both health-related applications and food preservation [14]. From a technological perspective, the high concentration of these bioactive compounds has promoted the valorization of pomegranate peel as a source of natural preservatives and functional ingredients capable of improving the oxidative stability and microbiological quality of food products [13,15]. Consequently, the recovery of these compounds through sustainable extraction strategies is essential to maximize the potential of this agro-industrial byproduct.

However, the efficient extraction of polyphenols from plant materials remains challenging because these compounds differ considerably in their chemical structure, polarity, and localization within plant tissues, while also interacting with other matrix components. Consequently, extraction efficiency depends not only on the plant source but also on the extraction technique and solvent employed [4]. Conventional methods for polyphenol extraction, such as maceration or Soxhlet extraction, often involve high energy consumption and long processing times, as well as the use of toxic organic solvents, such as methanol, which limits their application in food-related processes [16,17,18,19,20,21]. Therefore, the development of alternative green extraction technologies has been growing, with ultrasound-assisted extraction (UAE) being one of the most widely studied approaches due to its efficiency, reduced solvent consumption, and industrial scaling at a low cost [4,12,20,22]. Nevertheless, despite these advantages, UAE also presents some limitations that should be considered. Localized temperature increases generated by cavitation phenomena may promote the degradation of thermolabile phenolic compounds, while excessive sonication time or high ultrasonic intensity can induce oxidative degradation and compromise extract quality and reduce extraction yields [23,24]. Moreover, the heterogeneous distribution of ultrasonic energy within the extraction medium may affect process reproducibility, and scaling UAE from laboratory to industrial level remains challenging due to differences in energy distribution, equipment design, and process efficiency [25]. Therefore, careful selection of the extraction conditions is required to maximize the recovery of bioactive compounds while minimizing their degradation.

In parallel, the replacement of conventional organic solvents with safer and more suitable alternatives has become a key research focus [20]. In this regard, aqueous acetic acid-based systems such as vinegar represent a promising green solvent due to their biodegradability and GRAS (Generally Regarded As Safe) status [26]. Distilled vinegar, unlike fruit vinegars, lacks bioactive compounds but can reach a higher acetic acid content (>15–20%), which contributes to its antimicrobial functionality [27]. Moreover, distilled vinegar is the predominant type of vinegar used in the food industry, due to its sensory neutrality and higher acetic acid content, compared to common fruit vinegars [28,29,30,31]. On the other hand, the low pH of vinegar may provoke alterations in the texture and palatability of some foods. Thus, buffered vinegar resulting from acetic acid neutralization can satisfy market needs for new food ingredients without harming food products [32].

Another promising strategy involves the use of cyclodextrins (CDs) as green solubilizing agents due to their ability to form inclusion complexes with bioactive compounds, enhancing extraction efficiency [33,34]. Among them, α-cyclodextrin (αCD) is particularly attractive for food applications due to its regulatory acceptance as a novel food ingredient in Europe and its functionality as a carrier and solubilizing agent [33,35]. Previous studies have evaluated the use of acetone or ethanol mixtures acidified with acetic acid (0.5–10%), combined with UAE, for extracting phenolic and flavonoid compounds from dried mushrooms [36,37]. In addition, aqueous and non-aqueous solvents, including (1–5%) modified (2-hydroxypropyl βCD) or non-modified βCD, have been studied for the extraction of bioactive compounds from red grape pomace, freeze-dried pomegranate and dried powdered pomegranate, dried peach pomace, dried *Polygonum cuspidatum*, dried olive leaves and dried red beet roots [38,39,40,41,42,43,44].

Despite these advances, important limitations remain. Organic solvent-based extraction systems generally require solvent removal before food application, increasing processing costs and reducing sustainability. Although acidic aqueous media can improve the extraction of phenolic compounds, their high acidity may limit the direct incorporation of the resulting extracts into food products [44]. Furthermore, cyclodextrin-assisted extraction has mainly been investigated using water or hydroalcoholic solvents, whereas its combination with food-grade acidic solvents has received little attention. Consequently, there is still a need for extraction systems capable of efficiently recovering polyphenols while producing extracts that are directly compatible with food applications.

In this context, combining vinegar and αCD represents more than a novel solvent formulation. The proposed system integrates two complementary extraction mechanisms within a fully food-grade medium. Vinegar provides an acidic environment that promotes the release of phenolic compounds from the plant matrix while simultaneously contributing intrinsic antimicrobial functionality. At the same time, αCD can improve the aqueous solubility and stabilization of extracted phenolics through inclusion complex formation, potentially enhancing extraction efficiency without relying on organic solvents. In addition, comparing buffered and non-buffered vinegar may provide valuable insights into the influence of acidity on extraction performance while considering the technological requirements of food applications. Thus, this experimental work aimed to evaluate the use of UAE in combination with vinegar (buffered (NaOH and KHCO_3_ salts; 0.3–0.7% acetic acid)/non-buffered (9.6–19.4% acetic acid)), combined with αCD, as an alternative green strategy for the extraction of bioactive compounds from pomegranate peel byproduct. For that purpose, the extracts obtained were analyzed in terms of total phenolic content (TPC), total antioxidant capacity (TAC), antimicrobial activity, and targeted phenolic composition. This approach provides comparative insights into solvent performance while focusing on environmentally friendly extraction systems suitable for potential food applications.

## 2. Materials and Methods

### 2.1. Materials and Reagents

Liquid distilled vinegars (medium and high acetic acid contents; all of them supplied as non-buffered vinegars) supplied by JR Sabater S.A. (Murcia, Spain) were used as a solvent for the extraction of bioactive compounds. The vinegar was buffered with sodium (NaOH) or potassium salts (KHCO_3_). α-cyclodextrin (αCD) was obtained from Wacker Chemie AG (Burghausen, Germany). Methanol (99.9% purity) was purchased from PanReac AppliChem ITW Reagents (Barcelona, Spain). Gallic acid, Folin–Ciocalteu reagent (2 N), (±)-6-hydroxy-2,5,7,8-tetramethylchromane-2-carboxylic acid (Trolox, 97% purity) and 2,2-diphenyl-1-picrylhydrazyl (DPPH, 95% purity) were obtained from Sigma-Aldrich (St. Louis, MO, USA). Anhydrous sodium carbonate (Na_2_CO_3_) and sodium hydroxide (NaOH) were purchased from PanReac AppliChem ITW Reagents (Barcelona, Spain).

*Listeria monocytogenes* (STCC 4032) and *Salmonella enterica* (STCC 443) strains were acquired as lyophilized cultures from the Spanish Type Culture Collection (STCC, Valencia, Spain). Tryptic Soy Broth (TSB) and Plate Count Agar (PCA) microbiological media were acquired from Scharlab (Barcelona, Spain). Oxytetracycline dihydrate was obtained from Sigma-Aldrich (St. Louis, MO, USA).

Dehydrated pomegranate (*Punica granatum* L.) peel powder was supplied by the company Agrosingularity S.L. (Murcia, Spain). According to the supplier, the raw material was obtained from the Spanish cultivar Mollar de Elche and harvested during the 2022 season. The product had a final moisture content < 10%, a water activity of 0.44, and a nutritional composition of 53.0 g/100 g carbohydrates (including 19.2 g/100 g sugars), 31.0 g/100 g dietary fiber, 3.3 g/100 g protein, 1.4 g/100 g fat (0.4 g/100 g saturated fat), and 0.7 g/100 g salt, providing an energy value of 1257 kJ (300 kcal) per 100 g. Samples were received vacuum-packaged in embossed vacuum bags and stored at room temperature, protected from light, until use.

### 2.2. Morphological Characterization of Powdered Byproducts

#### 2.2.1. Particle Size Distribution

The particle size distribution of the powdered pomegranate peel byproduct was measured by the wet method using a laser diffraction particle size analyzer (Mastersizer 2000, Malvern Instruments; Malvern, UK) coupled to a Hydro 2000SM accessory unit. A refractive index of 1.50 was used for the sample and 1.33 for the dispersing agent, along with an absorption index of 0.1. For each measurement, D_[3,4]_ or X_vm_ (De Brouckere mean diameter; indicates the diameter of the particles that occupy most of the sample volume), D_[2,3]_ or X_sv_ (Sauter mean diameter; refers to the diameter of a particle whose average surface area corresponds to that of the entire distribution), D_10_, D_50_ and D_90_ (percentiles that represent the diameter of the particles below 10, 50 and 90% of the volume of the distribution, respectively) and Span parameters (designates the width of the particle size distribution and, therefore, the distance between D_10_ and D_90_ with respect to D_50_) were obtained [45,46].

#### 2.2.2. Scanning Electron Microscopy (SEM)

Morphology of the dehydrated pomegranate peel was examined using scanning electron microscopy (SEM). Black and white micrographs were recorded using a Hitachi S-3500N scanning electron microscope (Hitachi, Tokyo, Japan) with a tungsten filament electron source, automatic polarization and electronic alignment of the electron gun with an accelerating voltage of 0.5–30 kV and a magnification range of 18–300,000×.

### 2.3. Selection of the Ultrasound-Assisted Extraction Conditions

The evaluation of UAE conditions (1, 2 and 3; see next subheadings) was conducted using an ultrasound device Bandelin Sonorex Digiplus DL 514 BH (Berlin, Germany), according to the procedure described by Zain et al. [47], with some modifications. Briefly, sample preparation prior to UAE involved homogenizing the powdered byproduct with the corresponding solvent at a 1:50 ratio (*weight (w)*:*volume (v)*) at 3.000 rpm for 30 s (T 25 hidigital ULTRA-TURRAX^®^; Staufen, Germany). The 1:50 ratio (*w*:*v*) was selected consistently with preliminary experiments. Several solid-to-liquid ratios were explored within standard ranges reported in the literature for plant matrices [47]. A balanced s/L ratio was selected for the final experiments since we observed that outside this range, the extraction efficiency was suboptimal, as higher amounts of solvent improved diffusion but diluted the extract, while lower amounts limited solubilization capacity [22]. The UAE was performed at 35 kHz and 600 W of heating power (720 W peak ultrasonic power and 180 W nominal ultrasonic power). The chosen ultrasonic frequency and conditions applied here were in the range of 20–120 kHz, usually employed for the most effective extraction of bioactive compounds from fruit and vegetable byproducts [25]. In this study, ultrasonic parameters (power and frequency) were kept constant and standardized, based on previous experiments according to the scientific literature, to reach an efficient cavitation for plant matrices like the ones evaluated here, without degrading the bioactive compounds of interest. Dry extract yield was not evaluated, as the focus of this study was on the selective extraction and characterization of bioactive phenolic compounds rather than on global extraction yield. Owing to the massive diversity of individual polyphenols in plant sources, optimal conditions were selected, given the quantitative results of TPC and TAC of the extracts and not on the individual compound composition, prioritizing the selection of conditions that would allow obtaining extracts with a potentially high antioxidant composition.

#### 2.3.1. Experiment 1: Evaluation of Temperatures and Times for the Ultrasound-Assisted Extraction

The effect of different extraction times (15, 30, 60 min) and temperatures (20, 30, 60 °C) during UAE (without αCD in Experiment 1) was evaluated (Table 1). The selection of temperature and time ranges was guided by the specific literature and preliminary studies that identified adequate thresholds for maintaining a balance between extraction efficiency and compound stability [48]. For Experiment 1, the solvents studied were ultrapure water (W), an aqueous mixture of methanol–ultrapure water (1:1; *v*:*v*) (M50), and unbuffered vinegars (with high (≈20%) acetic acid content (V1) or moderate (≈10%) acetic acid content (V2)). The supernatant of the resulting liquid extract was assessed for the analysis of total phenolic content, antioxidant-related properties, and antimicrobial activity of the extracts.

#### 2.3.2. Experiment 2: Evaluation of Different αCD Concentrations for the Ultrasound-Assisted Extraction

After selecting the most suitable UAE time:temperature conditions, different αCD concentrations were studied, as described in Table 2. The concentration of a cosolvent (such as αCD) influences the solubility and selectivity of the target compounds and may impact dissolution and diffusion. The αCD concentrations studied were 10 or 15%, determined by preliminary studies, since the solubility limit of αCD at 25 °C is 14.5 g in 100 mL [34]. In addition to the previous solvents (W, M50, V1 and V2), a 100% methanol solvent (M100) was also studied based on the results from the previous experimental step. Methanol-based systems (50% and 100%) were included as reference extraction solvents widely used in phenolic extraction studies. Aqueous methanol allows polarity adjustment, while absolute methanol is commonly used as a benchmark for efficient extraction. These solvents were included for comparative purposes and not as solvents intended for food applications. pH/total titratable acidity values of V1, V1-10 and V1-15 were 2.8–2.9/18.7–19.4% and for V2, V2-10 and V2-15 were 3.0–3.1/9.6–9.7%. Again, total phenolic content and antioxidant-related properties of extracts were analyzed to evaluate the best extraction conditions.

#### 2.3.3. Experiment 3: Evaluation of the Use of Buffered/Unbuffered Vinegars for the Ultrasound-Assisted Extraction

Once the previous extraction conditions were selected, unbuffered (V) or buffered (sodium (VT1) or potassium salts (VT2)) vinegars were studied as solvents for pomegranate (P) byproduct powder (Table 3). The pH of the medium (unbuffered/buffered vinegar) can also affect the ionic state of bioactive compounds, their stability, and their interaction with the solvent. Adjusting between V and VT vinegar allows for exploring these effects and stabilizing the pH-sensitive compounds [25]. M50 was utilized as an extraction control solvent and 10% αCD based on the previous Experiment 2. pH/total titratable acidity values of V1, V1-10 and V1-15 were 2.8–2.9/18.7–19.4% and for V2, V2-10 and V2-15 were 3.0–3.1/9.6–9.7%. Total phenolic content, antioxidant-related properties and antimicrobial activity of the resultant extracts were then analyzed.

### 2.4. Conventional Extraction Procedure

Conventional extraction (CE) of polyphenols from dehydrated pomegranate peel byproduct was carried out using an orbital shaker (Stuart; Staffordshire, UK), following the methodology proposed by Chen and Yang [49], with slight adaptations. W and M50 solvents were used as control solvents. Unbuffered distilled vinegar was utilized as a green alternative solvent (V1, V2). Liquid solvents were mixed with the powdered dehydrated pomegranate byproduct (1:50 (*w*:*v*) powder:solvent ratio) at 3000 rpm for 30 s (T 25 digital ULTRA-TURRAX^®^; Staufen, Germany). Samples were then kept at 120 rpm for 1 h at room temperature and in dark conditions. Total phenolic content and antioxidant-related properties of the resulting extracts were evaluated.

### 2.5. Total Phenolic Content

Total phenolic content (TPC) of the extracts was determined according to the Folin–Ciocalteu’s method described by Singleton and Rossi [50], with some modifications. Firstly, resulting extracts were centrifuged at 14,000× *g* (10 min/4 °C) (Sorvall Legend Micro 17R, Thermo Scientific; Waltham, MA, USA), and the supernatant was used as the TPC extract. The TPC was determined by taking 19 µL of the TPC extract, which was placed in a well of a 96-well microtiter plate (Greiner Bio-One; Frickenhausen, Germany); 29 µL of 0.5 N Folin–Ciocalteu solution (2 N, Sigma-Aldrich; St. Louis, MO, USA) was then added and incubated for 3 min in the dark at room temperature. Subsequently, 192 µL of a 0.4% NaOH/2% Na_2_CO_3_ mix was added and allowed to incubate for 2 h in the dark at room temperature. Finally, the absorbance was measured at 750 nm using a microplate reader (Infinite M Plex 200 Pro, Tecan Trading AG; Männedorf, Switzerland). The TPC was expressed as mg of gallic acid equivalents (GAE) per g of dry weight (DW), using a gallic acid calibration curve (0–1 mM) for it. Each of the TPC extracts was analyzed in triplicate.

### 2.6. Total Antioxidant Activity

The total antioxidant capacity (TAC) of extracts was measured following the DPPH free radical scavenging assay, according to the method described by Brand-Williams et al. [51], with slight modifications. The same previous TPC extract was used as the TAC extract. The resulting supernatant after centrifugation at 14,000× *g* (10 min/4 °C) (Sorvall Legend Micro 17R, Thermo Scientific, USA) was used as the TAC extract. The TAC was determined by mixing 20 µL of the TAC extract with 194 µL of adjusted (Abs_515_ = 1.10 ± 0.02) DPPH reagent in a 96-well microtiter plate (Greiner Bio-One; Frickenhausen, Germany) [52]. Samples were then incubated for 25 min in the dark at room temperature. Finally, the absorbance was measured at 515 nm using the microplate reader. The TAC was expressed as µmol of TROLOX equivalents (TE) per g of DW, using a TROLOX calibration curve (0–1 mM) for it. Each of the TAC extracts was analyzed in triplicate.

### 2.7. HPLC-QTOF-MS Analysis

The analysis of phenolic compounds in pomegranate byproduct extracts was performed using high-performance liquid chromatography coupled with quadrupole time-of-flight mass spectrometry (HPLC-QTOF-MS; Agilent 1290 Infinity II LC system and Agilent 6550 QTOF MS, Agilent Technologies; Santa Clara, CA, USA). The method was adapted from previously reported procedures [14] and optimized to support the targeted selection and quantification of representative phenolic markers in pomegranate peel extracts.

Chromatographic separation was achieved using an Agilent ZORBAX Eclipse Plus C18 column (2.1 × 100 mm, 1.8 µm), maintained at 40 °C. The autosampler temperature was set at 5 °C, and the injection volume (20 µL) was optimized for analytical sensitivity. The mobile phase consisted of MilliQ water with 0.1% formic acid (A) and methanol (B), delivered at a flow rate of 0.4 mL/min under a gradient elution program optimized to achieve sufficient resolution of phenolic compounds.

Mass spectrometry detection was performed in negative electrospray ionization (ESI^−^) mode. The optimized parameters were set as follows: nebulizer gas pressure, 30 psi; drying gas flow, 16 L/min at 130 °C; sheath gas flow, 11 L/min at 300 °C; capillary voltage, 3000 V; nozzle voltage, 100 V; fragmentor voltage, 360 V; and octopole RF voltage, 750 V. Data were acquired over an *m*/*z* range of 50–1100 with an acquisition rate of 4 spectra/s. Continuous mass calibration was ensured using reference ions at *m*/*z* 112.9856 and 1033.9881. Data-independent acquisition (DIA) was carried out using the All Ions approach with collision energies of 0, 10, and 40 eV, enabling simultaneous acquisition of precursor and fragment ion information. Data processing was performed using MassHunter Qualitative Analysis Navigator software (Agilent Technologies, Rev. B.08.00).

Tentative identification of phenolic compounds was used exclusively as a supporting tool for marker selection and was based on accurate mass measurements (mass error < 5 ppm), MS/MS fragmentation patterns, retention time behavior, and comparison with literature data and spectral databases. This step was not intended to provide a comprehensive characterization of the phenolic profile, but rather to guide the selection of representative compounds for targeted quantification.

Quantification was performed using a strictly targeted approach restricted to selected phenolic markers for which authentic standards were available, including ellagic acid, gallic acid, catechin, and punicalagin (certified reference materials from commercial suppliers). This selection was based on the availability of authentic standards and the relevance of these compounds as representative phenolics in pomegranate peel [14,53]. Calibration curves were prepared at multiple concentration levels covering the expected range in samples and showed excellent linearity (R^2^ > 0.999). Limits of detection (LOD) and quantification (LOQ) were determined based on signal-to-noise ratios of 3 and 10, respectively. Quantification was carried out by integrating peak areas of the corresponding analytes observed in the chromatogram, using the most selective ion for each compound, and results were expressed as mg of compound per g of DW (mg/g DW).

Finally, the quantified phenolic compounds were used as chemical markers to comparatively characterize the phenolic profiles of extracts obtained with the different solvent systems studied. This targeted analytical approach was applied exclusively to selected marker compounds and does not constitute a full metabolomic or comprehensive profiling of all LC-QTOF-MS detectable signals. Therefore, non-quantified detected compounds were not included in the results, as they fall outside the scope of the targeted analytical design. This strategy enabled a more specific and reliable comparison of solvent selectivity and phenolic distribution than that obtained from global antioxidant assays such as DPPH and Folin–Ciocalteu.

### 2.8. Antimicrobial Activity of Extracts

Antimicrobial activity of extracts was evaluated by the Kirby–Bauer well-diffusion method [54], with adaptations. Firstly, lyophilized pathogenic strains *Listeria monocytogenes* STCC 4032 and *Salmonella enterica* STCC were initially activated according to STCC specifications in TSB (Tryptic Soy Broth), making two passages (37 °C/20 h per passage) to an approximate concentration of 10^8^ CFU/mL. Then, 0.1 mL of microorganism at a concentration of 10^6^ CFU/mL was inoculated onto PCA (Plate Count Agar) plates. Subsequently, three wells (7.0 ± 0.1 mm diameter) were performed on the solid agar. Then, 50 µL of extract (or control) was added to each corresponding well. Oxytetracycline dihydrate (100 mg/L, *w*:*v*) was used as a positive control, while sterilized distilled water represented the negative control. Plates were incubated at 37 °C for 24 h before measuring the diameter of the inhibition zone (mm) with a digital caliper. All determinations were performed in triplicate.

### 2.9. Statistical Analysis

Data were subjected to statistical analysis using RStudio software (v.4.2.3, Posit Software; Boston, MA, USA). Prior to ANOVA, data were evaluated to confirm their suitability for parametric analysis. Results were analyzed at a 95% confidence level (*p* < 0.05). Depending on the experimental design, one-way or two-way analysis of variance (ANOVA) was applied, followed by Tukey’s HSD test for multiple comparisons. Data are presented as mean values of three independent biological replicates ± standard deviation.

## 3. Results and Discussion

### 3.1. Morphological Characterization of the Raw Material

**Figure 1:**
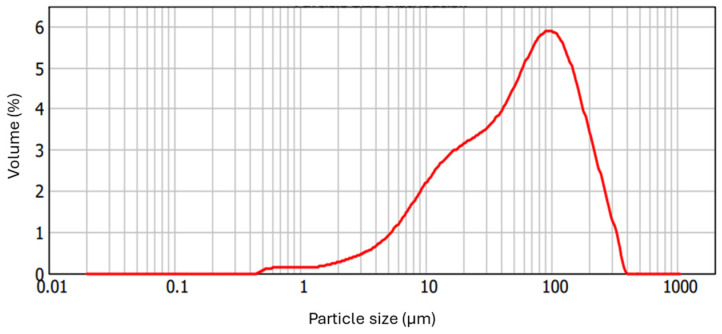
Particle size distribution (µm) of the studied powdered dehydrated pomegranate byproduct.

The powdered pomegranate peel was characterized in terms of particle size distribution and surface morphology to better understand its potential influence on the extraction of bioactive compounds. The particle size distribution (Figure 1) revealed a broad and heterogeneous profile, with D10, D50 and D90 values of 9.06, 57.93 and 181.89 µm, respectively. The span value (≈2.98) further confirmed the high polydispersity of the sample.

The presence of fine particles (<10 µm) suggested an increased specific surface area, favoring solvent penetration and the release of phenolic compounds during extraction [55,56]. Conversely, the larger particles represented by the D90 fraction may impose diffusion limitations under mass-transfer-restricted conditions. Overall, this heterogeneous particle size distribution indicates that extraction likely involved a combination of rapid solute release from fine particles and slower diffusion from larger fragments [56].

On the other hand, the volume-weighted mean particle size (D[3,4] = 78 µm) of the pomegranate peel powder was slightly lower than values reported in previous studies (>85 µm) [57], which may be attributed to differences in the grinding process applied. However, this value remains within the range reported for micronized pomegranate peel (41–142 µm) [58]. The relatively narrow and controlled particle size in the present study was intended to minimize the influence of particle size on mass transfer during extraction. In this way, diffusion-related limitations were reduced and kept comparable across all solvent systems, allowing a more direct evaluation of the effect of solvent composition on extraction efficiency. Although particle size was controlled, differences in solvent properties still govern mass transfer behavior during extraction [56,58].

**Figure 2:**
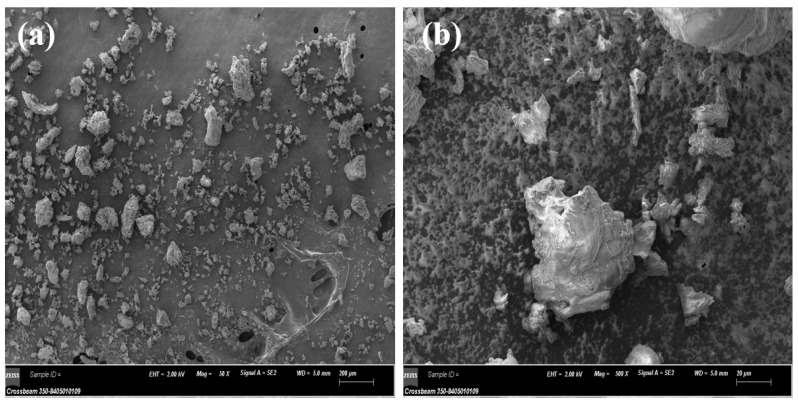
SEM micrographs of dehydrated powdered pomegranate at low (50×) (**a**) and higher magnification (500×) (**b**).

These observations were consistent with the morphological features observed by scanning electron microscopy (SEM) (Figure 2). At low magnification (50×) (Figure 2a), the sample exhibited a highly heterogeneous structure composed of irregularly shaped particles with a wide size distribution, including large fragments and fine particles forming aggregates. This visual evidence corroborated the broad particle size distribution results obtained by laser diffraction analysis. At higher magnification (500×) (Figure 2b), particles exhibited irregular fractured surfaces and rough morphologies, characteristic of mechanically milled pomegranate peel and consistent with previous reports [58,59].

The presence of surface irregularities and fractured structures is expected to enhance solvent accessibility by increasing the effective contact area between the solid matrix and the extraction medium. Overall, the combined particle size and SEM analysis confirmed that the pomegranate peel powder consisted of a heterogeneous and mechanically disrupted matrix, which provides favorable conditions for solvent accessibility and mass transfer during extraction. Since the same powdered material was used in all experiments, these morphological characteristics remained constant throughout the study. Therefore, the differences observed in extraction performance among treatments can be primarily attributed to the extraction solvent composition rather than to variations in the physical properties of the plant matrix, allowing a more reliable comparison of the extraction systems evaluated in this study.

### 3.2. Effect of UAE Operating Parameters on Extraction Performance

#### 3.2.1. Effect of Time

**Figure 3:**
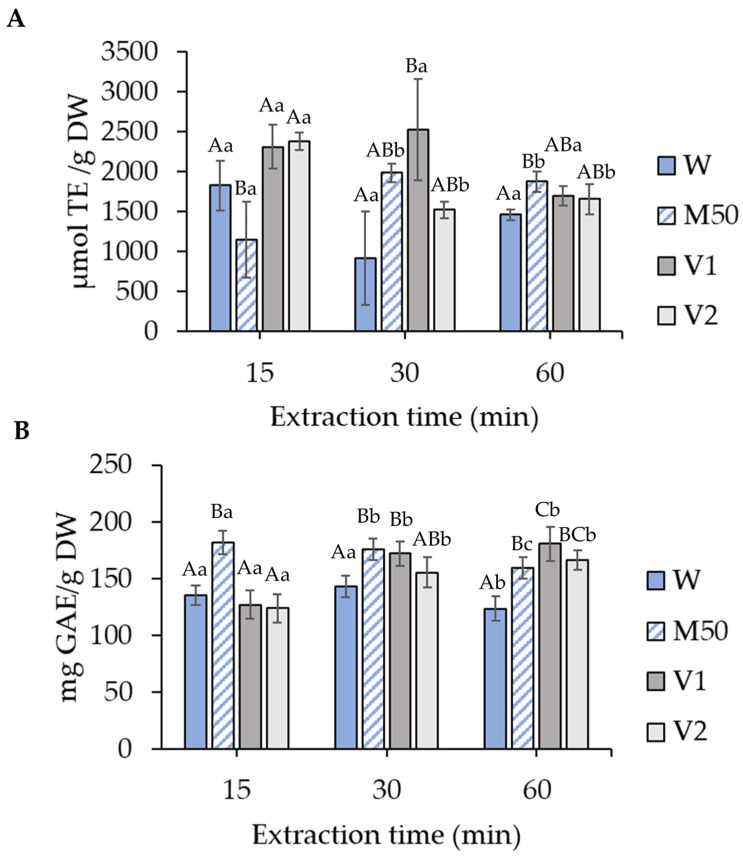
Effect of UAE time on TAC (µmol TE/g DW) (**A**) and TPC (mg GAE/g DW) (**B**) of pomegranate byproduct powder (mean (*n* = 3) ± standard deviation). W = ultrapure water; M50 = methanol–ultrapure water (1:1); V1 = distilled vinegar with a high acetic acid content; V2 = distilled vinegar with a moderate acetic acid content. Different uppercase letters indicate significant differences (*p* < 0.05) among extraction solvents for the same extraction time. Different lowercase letters indicate significant differences (*p* < 0.05) among extraction times for the same extraction solvent, according to two-way ANOVA followed by Tukey’s HSD test.

Figure 3 shows the effect of extraction time on the total antioxidant capacity (TAC) (Figure 3A) and total phenolic content (TPC) (Figure 3B) for the different solvent systems. In general, extraction time influenced both responses, although the magnitude and direction of these effects were solvent dependent.

Differences were found in TAC of the extracts among the extraction times. M50 extracts showed an increase when the extraction time was extended from 15 to 30 and 60 min, suggesting a progressive release of antioxidant compounds under these conditions. In contrast, W and V1 extracts did not show notable differences between 15 and 30 min, which may indicate that near-equilibrium conditions were reached at relatively short extraction times for these solvents. Interestingly, V2 extracts showed a decrease in TAC with increasing extraction time (>30 min), possibly associated with instability or transformation of antioxidant compounds under prolonged ultrasound exposure.

Regarding TPC, vinegar-based extracts (V1 and V2) exhibited an increase after 60 min of extraction, while W and M50 extracts showed a slight decrease at longer extraction times. This suggests that, although extended extraction time may favor the release of phenolic compounds in acidic media, prolonged sonication may also be associated with changes in the composition or stability of antioxidant compounds, as previously reported for ultrasound-assisted extraction of phenolic-rich matrices, such as anthocyanins present in pomegranate peel [11,13,25].

The initial extraction stage (15 min) appeared to be dominated by rapid cavitation-assisted mass transfer, where ultrasound facilitates solvent penetration and cell disruption, leading to fast release of bioactive compounds. This behavior is consistent with previous studies reporting that a large fraction of phenolic compounds is extracted during the early stages of UAE in different plant matrices like blackberries, but then followed by a decrease when approaching equilibrium from 30 to 60 min [60].

At longer extraction times (>30 min), a balance between continued extraction and potential degradation phenomena may occur. Solvent saturation, oxidation reactions and ultrasound-related degradation reactions have been reported to reduce antioxidant responses when extraction is prolonged [43,47].

In particular, the decrease in TAC observed for V1 and V2 extracts after 30 min may be related to the instability of certain antioxidant compounds under prolonged ultrasound exposure, despite the protective effect of acidic conditions. Although low pH can reduce oxidation reactions, extended sonication may still promote structural changes in sensitive molecules [44,47]. Similar trends have been reported in other studies, where prolonged ultrasound extraction did not further increase phenolic recovery and was associated with reduced antioxidant parameters [47,61].

From a process perspective, our results suggested that extraction time influences both extraction efficiency and compound stability. Short extraction times (15–30 min) appear sufficient to achieve substantial recovery of antioxidant compounds from pomegranate peel byproduct, whereas longer extraction times may not further improve extraction and may be associated with changes in the stability or composition of the obtained extracts [43,62].

#### 3.2.2. Effect of Temperature

**Figure 4:**
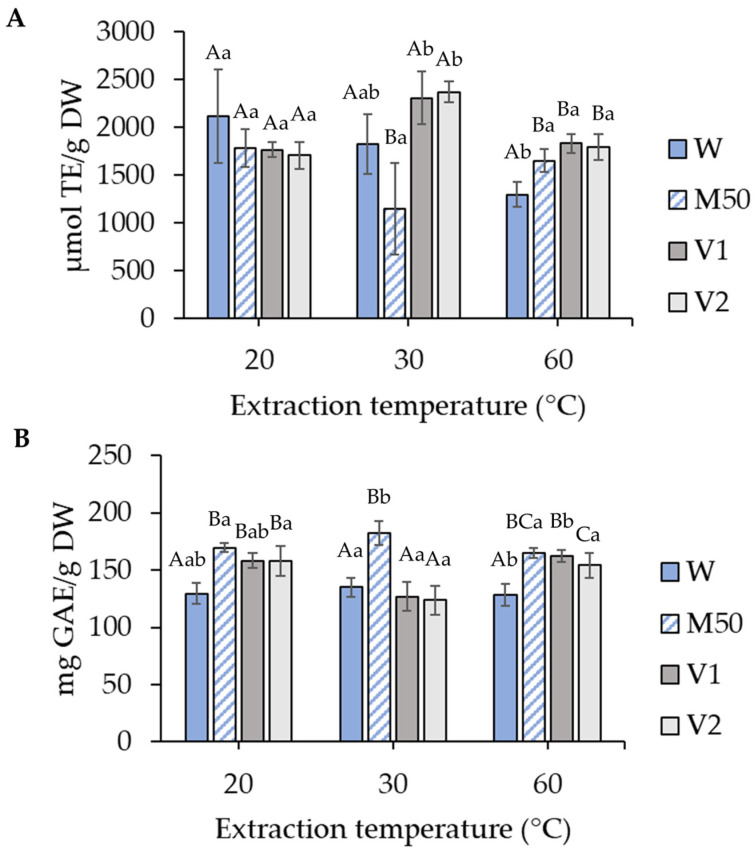
Effect of UAE temperature on TAC (µmol TE/g DW) (**A**) and TPC (mg GAE/g DW) (**B**) of pomegranate byproduct powder (mean (*n* = 3) ± standard deviation). W = ultrapure water; M50 = methanol–ultrapure water (1:1); V1 = distilled vinegar with a high acetic acid content; V2 = distilled vinegar with a moderate acetic acid content. Different uppercase letters indicate significant differences (*p* < 0.05) among extraction solvents for the same extraction temperature. Different lowercase letters indicate significant differences (*p* < 0.05) among extraction temperatures for the same extraction solvent, according to two-way ANOVA followed by Tukey’s HSD test.

The effect of temperature on the extraction of antioxidant compounds from pomegranate byproduct is shown in Figure 4. According to different authors, increasing temperature during UAE generally follows a similar trend to that observed with increasing extraction time [43,60]. In the present study, increasing the extraction temperature from 20 to 30 °C led to a significant increase in the TAC of V1 and V2 extracts (Figure 4A). In contrast, no differences were observed for W and M50 extracts when the temperature increased, although a significant reduction in TAC was detected for the W extract at 60 °C. Similarly, TPC of extracts showed a slight, non-significant (*p* > 0.05) increase (Figure 4B).

An increase of 10 °C in UAE temperature appeared to be sufficient to enhance the solubility of polyphenols in the extraction solvents, which was particularly noticeable in the M50 extracts [25,60]. Thus, the use of 30 °C extraction temperature may have contributed to a more efficient extraction of antioxidant compounds, likely due to improved solubility combined with a possible reduction in the viscosity of the solvent–solute system, thereby facilitating the mass transfer of polyphenols into the liquid phase [25]. However, a significant reduction in TPC was observed in the M50 extracts at 60 °C, which may be attributed to the polymerization of these compounds or to a decrease in solvent surface tension, potentially limiting the transfer of polyphenols into the liquid phase [25,43,44]. These findings are consistent with previous studies reporting that UAE at 60 °C decreased total polyphenol and total flavonoid contents in oil palm leaf extracts [47].

The application of short extraction times combined with moderate temperatures might have favored the enrichment of polyphenols in V1 and V2 extracts, resulting in extracts with comparatively higher antioxidant capacity than M50 extract [62]. These results are in agreement with other studies suggesting a UAE temperature range of 25–35 °C as suitable for polyphenol extraction from pomegranate peel [61]. Nevertheless, other authors have reported increased polyphenol yields from blueberries at higher ultrasonic temperatures (60 °C) [60]. These differences highlight the need to adapt extraction conditions to the specific plant matrix.

It should be noted that, in general, extraction temperatures above 45 °C tend to be associated with a reduction in the yield of bioactive compounds, likely due to the degradation of heat-sensitive compounds, such as anthocyanins and certain phenolic compounds [25,44,47]. Additionally, the combination of V1 and V2 solvents with high temperatures (60 °C) may have promoted the formation of compounds capable of absorbing at similar wavelengths to polyphenols, which could explain the apparent increase in TPC despite the lower TAC observed under these conditions [47]. Therefore, under the conditions evaluated in this study, 30 °C can be considered an appropriate temperature for UAE of dehydrated pomegranate peel byproduct, as it allowed a favorable balance between extraction efficiency and preservation of antioxidant compounds. Furthermore, although higher temperatures may enhance polyphenol extraction, they may also induce the degradation of other thermolabile bioactive compounds, such as natural antimicrobial agents, potentially reducing their functional properties [63].

#### 3.2.3. Effect of Solvent and Cyclodextrin Concentration

**Figure 5:**
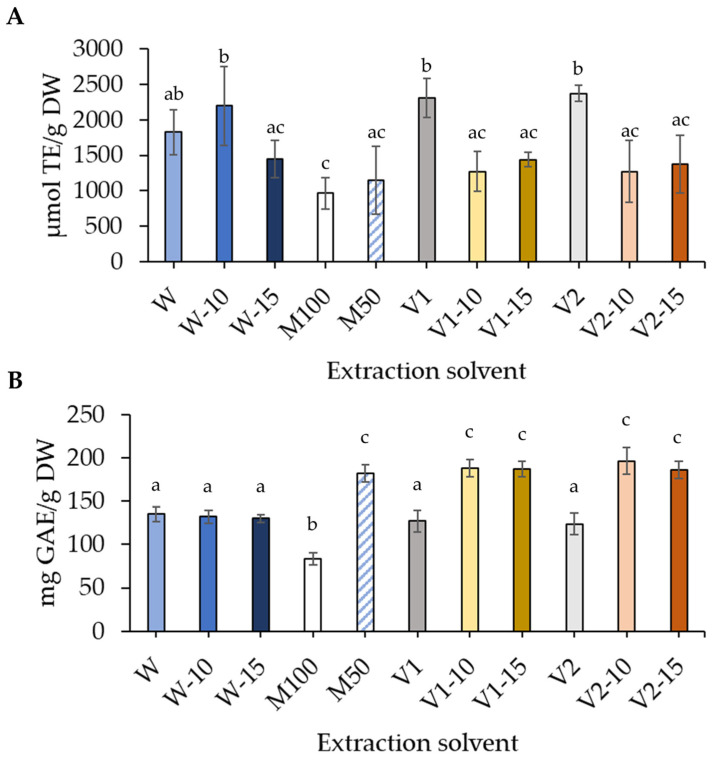
Effect of UAE solvent and cyclodextrin concentration on TAC (µmol TE/g DW) (**A**) and TPC (mg GAE/g DW) (**B**) of pomegranate byproduct powder (mean (*n* = 3) ± standard deviation). W = ultrapure water; W-10 = W + 10% αCD; W-15 = W + 15% αCD; M100 = absolute methanol; M50 = methanol–ultrapure water (1:1); V1 = distilled vinegar with a high acetic acid content; V1-10 = V1 + 10% αCD; V1-15 = V1 + 15% αCD; V2 = distilled vinegar with a moderate acetic acid content; V2-10 = V2 + 10% αCD; V2-15 = V2 + 15% αCD. Different letters indicate significant differences (*p* < 0.05) among treatments, according to one-way ANOVA followed by Tukey’s HSD test.

Figure 5 presents the antioxidant properties (TAC, Figure 5A; TPC, Figure 5B) of pomegranate extracts obtained by UAE (30 °C, 15 min, 100% power) using aqueous and organic (methanol) solvents, as well as unbuffered distilled vinegar (V1 and V2), with the incorporation of various concentrations of αCD (0–15%). As observed, the use of water or solvent systems containing an aqueous fraction generally resulted in higher TAC values (Figure 5A). No differences were found between W and M50 solvents. In contrast, absolute methanol (M100) (966.8 ± 220.9 µmol TE/g DW) showed significantly lower TAC values compared to W extract (1824.9 ± 313.9 µmol TE/g DW), whereas no differences (*p* > 0.05) were observed when compared to M50 (1147.5 ± 477.6 µmol TE/g DW). This behavior may be related to the predominantly hydrophilic nature of antioxidant compounds present in the pomegranate byproduct, which may exhibit greater solubility in polar solvents (W, M50, vinegar and vinegar-αCD systems) than in less polar ones such as M100 [47].

Vinegar-based solvents (V1 and V2) showed the highest TAC, with no differences (*p* > 0.05) when compared to the W-10 solvent. The recovery of certain compounds, such as pectin from fruit and vegetable matrices, has been reported to be enhanced under acidic conditions (pH = 1–5) [25], which may partially explain these results. Acetic acid content did not appear to significantly affect TAC between V1 (2309.8 ± 273.5 µmol TE/g DW) and V2 (2372.1 ± 109.7 µmol TE/g DW), as no differences (*p* > 0.05) were observed. However, it is worth noting that the solubilization of dehydrated pomegranate byproduct and αCD seemed to be improved when moderate acetic acid content was used (V2), compared to V1. The incorporation of αCD did not significantly enhance TAC values, as no differences (*p* > 0.05) were observed between W and vinegar solvents and their corresponding αCD-containing systems (10–15%). Nevertheless, all aqueous-based systems showed higher TAC than M100, supporting the potential of these solvents as alternatives to conventional organic solvents such as methanol.

Solvent polarity also appeared to influence TPC (Figure 5B). The higher polarity of water resulted in significantly higher TPC values compared to M100 (134.9 ± 8.5 mg GAE/g DW *vs* 83.4 ± 6.6 mg GAE/g DW, *p* < 0.05). However, the use of an aqueous methanol mixture (M50) (182.0 ± 10.4 mg GAE/g DW) led to significantly higher TPC compared to W (*p* < 0.05). Both water and methanol are commonly used solvents for polyphenol extraction, and the polarity of water may facilitate the extraction of hydrophilic bioactive compounds from plant matrices [63]. No differences were observed between water (134.9 ± 8.5 mg GAE/g DW) and vinegar extracts (126.9 ± 12.6 mg GAE/g DW for V1; 123.6 ± 12.8 mg GAE/g DW for V2). Both V1 and V2 extracts showed significantly higher TPC than M100, which may be attributed to their higher polarity and the presence of acetic acid. Given that polyphenols are generally hydrophilic, polar solvents are typically more suitable for their extraction from plant byproducts [63,64]. Vinegar, being an aqueous solution containing approximately 3–5% (*w*:*v*) acetic acid (>95% water) [65], provides a favorable medium for the extraction of water-soluble polyphenols.

The incorporation of αCDs into W extracts (W-10 and W-15) did not result in significant differences (*p* > 0.05) in TPC, presumably due to a slight decrease in effective polarity of the solvent system. In contrast, vinegar systems appeared to benefit from αCD addition. Cyclodextrins are water-soluble due to their hydrophilic conformation on the surface [35], although acidic conditions may promote partial hydrolysis. In the case of vinegar, the combination of high-water content and αCD may have contributed to a more stable system, potentially through partial inclusion of acetic acid within the hydrophobic cavity of αCD, which could influence solvent properties [34,35]. The ability of CDs to form inclusion complexes is particularly relevant for polyphenol extraction, as it may enhance the apparent solubility of these compounds [33].

No differences (*p* > 0.05) were observed between vinegar–αCD systems and M50, suggesting that the combinations evaluated in this study could represent potential alternatives to methanol-based systems for polyphenol extraction from plant byproducts [33]. The addition of αCD to vinegar (V1 and V2) may have slightly modified polarity and enhanced polyphenol solubility through partial inclusion phenomena during UAE. Similar effects have been reported for other compounds, such as cyclosporine A, whose water solubility increased substantially in the presence of αCD due to complex formation [66]. Furthermore, no differences (*p* > 0.05) were observed between the use of 10% and 15% of αCD. TPC values of V1-10 (188.2 ± 9.8 mg GAE/g DW) and V2-10 (196.2 ± 15.6 mg GAE/g DW) were comparable to those of V1-15 (187.0 ± 9.4 mg GAE/g DW) and V2-15 (186.2 ± 9.9 mg GAE/g DW). These results suggest that increasing αCD concentration above 10% may not provide additional benefits under the conditions studied. The slightly lower TPC values observed at 15% αCD could be related to solubility limitations (14.5 g/100 mL) [34], which may have affected the effective interaction between αCD and polyphenols.

The results obtained in this study indicate that the effect of αCD on phenolic extraction was solvent-dependent. While αCD did not significantly increase TAC, it enhanced TPC in vinegar-based systems and achieved phenolic recoveries comparable to those obtained with the methanolic reference solvent. These findings suggest that αCD acts as a complementary extraction aid whose effectiveness depends on the extraction medium rather than as a universal enhancer of extraction efficiency. The enhanced UAE of polyphenols using αCD as an adjuvant observed in this study is in concordance with previous reports supporting the use of CDs in green extraction processes. Aqueous βCD systems (1–5%) have been shown to improve the extraction of polyphenols and other bioactive compounds from plant matrices such as beetroot [38], peach pomace [40], *P. cuspidatum* [41], red grape pomace [44], and pomegranate [42,43]. In addition, non-native CDs, such as 2-hydroxypropyl βCD (HP-βCD), have been successfully applied in aqueous systems or in combination with solvents such as glycerol or ethanol [38,39,42]. However, the use of additives βCD and γCD in foods is restricted in Europe, and the use of some derivatives (e.g., HP-βCD) is not authorized as food ingredients, although they are used as pharmaceutical excipients [33]. In contrast, αCD was selected in the present study because it is authorized as a Novel Food and can be incorporated into food applications as a carrier, stabilizer, solubilizer and dietary fiber. Although βCD and hydroxypropyl-βCD have frequently been reported as efficient extraction aids for phenolic compounds, the objective of this work was to develop a food-compatible extraction system based exclusively on ingredients suitable for potential food applications [33,35]. For this reason, the incorporation of αCD into vinegar-based solvent systems may represent a feasible strategy to support food-compatible extraction processes under the evaluated conditions, although its effect on extraction performance was solvent-dependent [33].

No differences were observed between V1 and V2 in terms of extraction efficiency, although TPC values were slightly higher in V2 + αCD extracts (186.2–196.2 mg GAE/g DW) compared to V1 + αCD extracts (187.0–188.2 mg GAE/g DW). While acidity did not significantly influence extraction yield, it appeared to affect solubilization and handling properties. For this reason, V2 was considered a more suitable extractant under the evaluated conditions. In addition, given the absence of differences between 10% and 15% αCD, the lower concentration (10%) may represent a more practical and economically favorable option.

#### 3.2.4. Effect of Ultrasound

**Figure 6:**
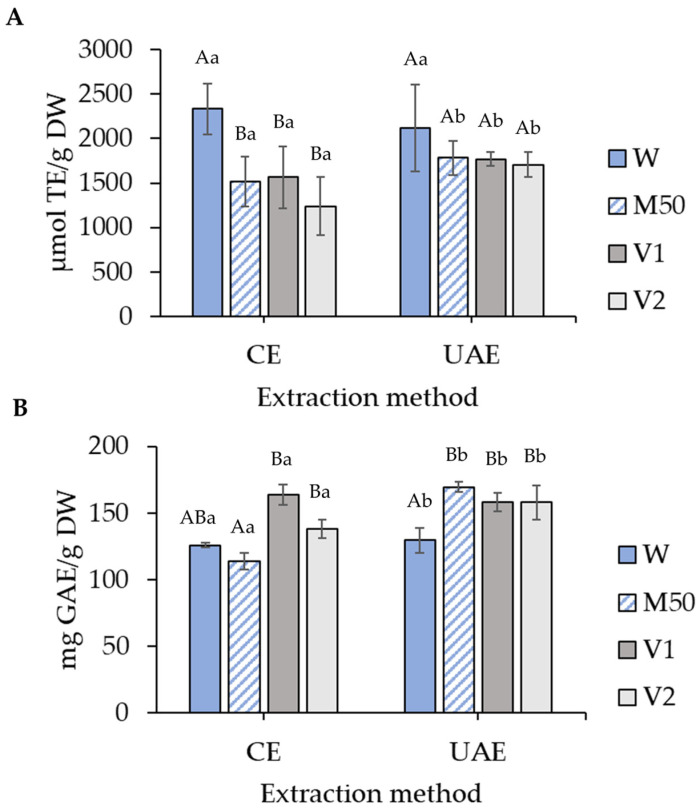
Effect of ultrasound on TAC (µmol TE/g DW) (**A**) and TPC (mg GAE/g DW) (**B**) of pomegranate byproduct powder (mean (*n* = 3) ± standard deviation). CE = conventional extraction (20 °C, 60 min, 120 rpm); UAE = ultrasound-assisted extraction (20 °C, 15 min, 100% power); W = ultrapure water; M50 = methanol–ultrapure water (1:1); V1 = distilled vinegar with a high acetic acid content; V2 = distilled vinegar with a moderate acetic acid content. Different uppercase letters indicate significant differences (*p* < 0.05) among extraction solvents for the same extraction method. Different lowercase letters indicate significant differences (*p* < 0.05) among extraction methods for the same extraction solvent, according to two-way ANOVA followed by Tukey’s HSD test.

The effect of applying ultrasound to the extraction of polyphenols from dehydrated pomegranate peel byproduct was explored using different extraction solvents (W, M50, V1 and V2), comparing the TAC (Figure 6A) and TPC (Figure 6B) of extracts obtained by conventional extraction (CE) (20 °C, 60 min, 120 rpm) and UAE (20 °C, 15 min, 100% power). Overall, TAC and TPC values obtained by UAE were significantly higher than those achieved by CE, despite the shorter extraction time (15 min *vs* 60 min). In addition, a higher TPC appeared to be associated with increased TAC, as widely reported in the literature for both sonicated and macerated extracts [67,68]. The TAC values obtained for pomegranate peel byproduct extracts after CE (1236.9–2334.1 µmol TE/g DW) were higher than those reported by other authors for pomegranate juice, wine and vinegar (10.04–13.39 mmol TE/L extract) [69].

Differences (*p* < 0.05) were observed between the TPC of V1 and V2 extracts (137.9–163.8 mg GAE/g DW) and those obtained using control solvents W (125.7–129.4 mg GAE/g DW extract) and M50 (113.8–169.5 mg GAE/g DW). The use of vinegar-based solvents appeared to favor polyphenol extraction in both CE and UAE procedures. However, no differences (*p* > 0.05) were observed between these extracts and M50 under UAE conditions. In general, the UAE resulted in higher TPC values in the liquid extracts while requiring shorter extraction times, suggesting a more efficient extraction process under the evaluated conditions. Similar trends have been reported by other authors, who observed higher extraction rates, yields and phenolic concentrations when applying continuous or pulsed ultrasound compared to CE [70]. In the same study, continuous ultrasound was found to be more effective at shorter extraction times than both pulsed UAE and CE.

Other studies have reported maximum yields in the UAE of polyphenols from pomegranate peel when applying a frequency of 20 kHz for 10 min, whereas traditional maceration or Soxhlet extraction generally required longer extraction times (up to 240 min) and resulted in lower yields [61,71,72]. In the present study, the results are in line with these observations, as shorter extraction times may have contributed to reduced degradation of bioactive compounds, thereby favoring higher TPC values in the liquid extracts [43,47,70].

The application of ultrasound at 35 kHz may have promoted the formation of cavitation bubbles of moderate size and number, which could facilitate mass transfer between the pomegranate peel matrix and the extraction solvent. The collapse of these bubbles has been reported to generate localized increases in temperature and pressure, which may contribute to enhancing the release of polyphenols into the liquid phase compared to non-sonicated systems [24,25,60]. In addition, sonication likely improved the contact between plant tissue and solvent, as cavitation has been widely reported to promote solvent penetration and is generally associated with increased mass transfer and possible cell wall disruption, thereby facilitating the diffusion of intracellular compounds [60]. These effects are consistent with the role of cavitation reported in the literature and may help explain the higher TAC and TPC values obtained compared to CE, where extraction is mainly driven by agitation. These results are consistent with previous studies reporting higher yields of total polyphenols and total flavonoids when applying an ultrasonic frequency of 40 kHz compared to conventional maceration at 120 rpm in plant matrices such as *Elaeocarpus serratus* [49]. The comparative results obtained in this study indicated that the extraction of bioactive compounds is influenced not only by the extraction technique but also by factors such as solvent composition, temperature and extraction time. Moreover, the UAE is generally considered a more environmentally friendly alternative to conventional maceration, as it may reduce extraction time and energy consumption, thereby supporting the use of more sustainable extraction approaches.

### 3.3. Alternative UAE of Polyphenols from Pomegranate Peel Byproduct

#### 3.3.1. Total Phenolic Content and Antioxidant Capacity

**Figure 7:**
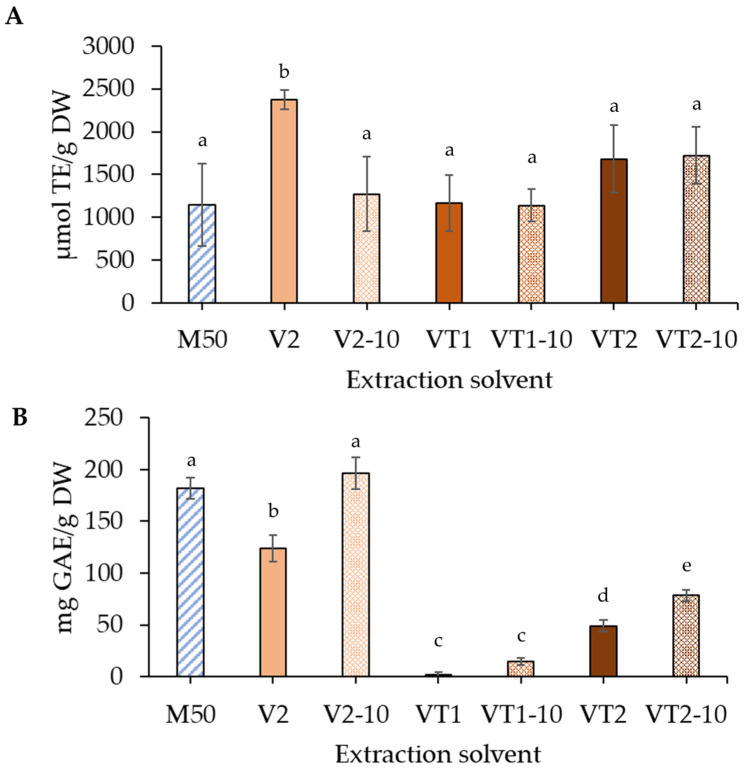
TAC (µmol TE/g DW) (**A**) and TPC (mg GAE/g DW) (**B**) of pomegranate byproduct powder after UAE (30 °C, 15 min, 100% power) (mean (*n* = 3) ± standard deviation). M50 = methanol–ultrapure water (1:1); V2 = distilled vinegar with moderate acetic acid content; V2-10 = V2 + 10% αCD; VT1 = sodium-buffered distilled vinegar; VT1-10 = VT1 + 10% αCD; VT2 = potassium-buffered distilled vinegar; VT2-10 = VT2 + 10% αCD. Different letters indicate significant differences (*p* < 0.05) among treatments, according to one-way ANOVA followed by Tukey’s HSD test.

The antioxidant-related properties of the extracts obtained with the different solvent systems were comparatively evaluated through total antioxidant capacity (TAC) and total phenolic content (TPC) assays (Figure 7). Differences among solvent systems were observed for both parameters, indicating that solvent composition influenced the extraction behavior of antioxidant-related compounds from pomegranate byproduct powder during UAE. In general, the non-buffered vinegar system (V2) showed the highest TAC values, whereas buffered vinegar systems exhibited more variable responses depending on the buffering salt and the presence of αCD. No differences (*p* > 0.05) in TAC were observed between the control (M50) and most vinegar-based systems, except for V2, which exhibited significantly higher TAC values (Figure 7A).

The relationship between TAC and TPC was only partial, suggesting that the antioxidant behavior of the extracts could not be exclusively determined by the total phenolic content estimated by the Folin–Ciocalteu assay. Although TPC is commonly associated with antioxidant-related properties in plant extracts, Folin–Ciocalteu is a global spectrophotometric assay that may also respond to other reducing compounds present in the extracts. Thus, the comparatively high TAC values observed in some vinegar-based extracts may reflect the contribution of additional antioxidant-related constituents co-extracted under acidic conditions [23]. Similar partial correlations between TAC and TPC have previously been reported in plant-derived extracts rich in heterogeneous antioxidant compounds [73,74,75].

The non-buffered vinegar systems (V2 and V2-10) generally showed higher TPC values than the buffered vinegar extracts, suggesting that acidic extraction conditions may favor the recovery and/or preservation of phenolic compounds during UAE (Figure 7B). Acidified extraction media have previously been associated with enhanced polyphenol solubilization, improved mass transfer, and reduced degradation of pH-sensitive phenolic compounds [36]. The use of acidified water and aqueous mixtures of acidified ethanol (8.5–10% acetic acid) as solvents proved to be effective in the extraction of polyphenols from *Boletus edulis* (3.73 and 3.42 mg GAE/g DW, respectively), compared to the use of hexane and diethyl ether (0.48 and 0.79 mg GAE/g DW, respectively) [37]. Similar observations have been reported for the extraction of anthocyanins and phenolic compounds from grape pomace and other plant matrices using acidified solvents [24,62,76]. In the present study, the marked reduction in TPC observed in buffered vinegar systems, particularly VT1, further supports the relevance of solvent acidity in the extraction behavior of pomegranate phenolics.

The influence of buffering differed depending on the salt composition of the solvent system. Sodium-buffered vinegar (VT1) showed the lowest TPC values among the studied extracts, whereas potassium-buffered vinegar systems (VT2 and VT2-10) maintained comparatively higher TAC despite reduced TPC values. These observations suggest that solvent composition and pH may differently influence the extraction of compounds contributing to antioxidant capacity and those detected by the Folin–Ciocalteu assay. Other studies have also highlighted the importance of solvent physicochemical properties in determining the selectivity of UAE towards different classes of bioactive compounds [19].

The addition of αCD led to solvent-dependent effects on TPC. In both buffered vinegar systems, αCD incorporation was associated with increased TPC values compared to corresponding solvents without cyclodextrin, while a more moderate increase was observed in the non-buffered vinegar system. This is because the use of cyclodextrins likely improves the apparent solubility and stabilization of phenolic compounds due to the formation of an inclusion complex, especially relevant in aqueous media [43]. In addition, acidic conditions may favor hydrophobic interactions between phenolic compounds and the CD cavity, potentially contributing to the differences observed among solvent systems. Nevertheless, the magnitude of these effects appeared to depend on the overall solvent composition and extraction conditions applied in the present study. The differences observed among solvent systems support the use of complementary analytical approaches to comparatively evaluate solvent-dependent variations in extract composition and antioxidant-related characteristics. However, it is acknowledged that antioxidant capacity was evaluated only using DPPH and Folin–Ciocalteu assays. The inclusion of additional assays (e.g., ABTS, FRAP) would provide a more comprehensive assessment and should be considered in future studies. Therefore, the observed differences should be interpreted as variations in DPPH radical scavenging capacity rather than as a comprehensive evaluation of the overall antioxidant activity of the extracts.

#### 3.3.2. Targeted Phenolic Composition (HPLC-QTOF-MS)

Since spectrophotometric assays such as Folin–Ciocalteu and DPPH provide global estimations of antioxidant-related properties, targeted HPLC-QTOF-MS analysis was further conducted to comparatively characterize representative phenolic compounds among the studied solvent systems. The targeted HPLC-QTOF-MS quantification revealed solvent-dependent differences in the distribution of the selected phenolic markers extracted from pomegranate byproduct powder after UAE (Table 4). Among the quantified compounds, punicalagin was the predominant phenolic marker in all extracts, representing the major proportion of the quantified phenolic fraction. This observation is consistent with previous studies describing α- and β-punicalagins as the principal ellagitannins present in pomegranate peel and among the major contributors to its antioxidant properties [14,77]. The quantified punicalagin concentrations obtained in the present work (26.1–64.5 mg/g DW) were within the range previously reported for pomegranate peel extracts (28.03–104.14 mg/g DW), although differences among studies are expected due to cultivar variability, extraction conditions, and analytical methodology [14,15].

The solvent system notably influenced the recovery of the targeted phenolic compounds. In general, the non-buffered vinegar solvent (V2), characterized by a moderate-to-high acetic acid content (9.6%), showed higher concentrations of gallic acid, catechin, punicalagin and total quantified phenolic markers than the control (M50) and the buffered solvents. In particular, V2 and V2-10 exhibited the highest sum of quantified phenolic markers (71.6 and 76.2 mg/g DW, respectively), whereas M50 reached 62.5 ± 10.7 mg/g DW. Similarly, punicalagin concentrations were higher in V2-based extracts (61.3–64.5 mg/g DW) than in M50 (51.7 ± 10.1 mg/g DW). These results suggest that acidic vinegar-based systems may favor the extraction and/or stabilization of representative pomegranate phenolics under UAE conditions applied in this study [53].

The observed differences between non-buffered and buffered vinegar systems indicate that extraction medium acidity likely played an important role in the recovery of phenolic compounds. Buffered solvents (VT1 and VT2), containing acetic acid concentrations of 0.3–0.7%, generally showed lower concentrations of the quantified markers, particularly in the potassium-buffered system (VT2). The sum of quantified phenolic markers decreased from 71.6 mg/g DW in V2 to 55.9 mg/g DW in VT1 and 29.2 mg/g DW in VT2. Similar trends were observed for punicalagin, ellagic acid, and catechin. Acidic conditions have previously been associated with improved polyphenol solubility and enhanced stability of hydrolyzable tannins during extraction, whereas increasing pH may promote degradation, oxidation, or reduced extractability of sensitive phenolic compounds [14,53]. The lower phenolic recovery observed in buffered systems may therefore be partially associated with pH-dependent changes affecting phenolic stability and solvent–solute interactions during UAE.

The incorporation of αCD produced solvent-dependent effects on the quantified phenolic compounds. In the unbuffered vinegar systems, αCD addition was associated with moderate increases in the sum of quantified markers and punicalagin concentration. More pronounced effects were observed in buffered systems, particularly in VT2, where the sum of quantified phenolic markers increased from 29.2 to 47.1 mg/g DW after αCD incorporation. In the same system, punicalagin concentration increased from 26.1 to 47.1 mg/g DW, while ellagic acid increased from 0.4 to 1.3 mg/g DW. Cyclodextrins have previously been reported to improve the apparent solubility and stabilization of phenolic compounds through inclusion complex formation, especially in aqueous systems [33]. Thus, the results presented here suggest that αCD may contribute to partially preserving or stabilizing representative phenolic compounds under less favorable extraction conditions.

The quantified concentrations of gallic acid and catechin obtained in the present work were also comparatively higher than several previously reported values for pomegranate peel extracts. Gallic acid concentrations ranged from 2.0 to 6.3 mg/g DW, whereas catechin ranged from 0.7 to 3.0 mg/g DW. Previous studies have reported substantially lower concentrations for these compounds (0.01–0.27 mg/g DW, and 0.61 mg/g DW, for gallic acid and catechin, respectively) depending on extraction methodology and plant material characteristics [14,78]. In contrast, the ellagic acid concentrations obtained here (0.4–2.3 mg/g DW) were generally within the ranges previously described in the literature (0.23–4.51 mg/g DW) [14,15,78]. These differences further support the strong influence of solvent composition and extraction conditions on the selective recovery of individual phenolic compounds.

Overall, the targeted quantification approach allowed comparative characterization of representative phenolic markers among the solvent systems studied, complementing the information obtained through global spectrophotometric assays (TPC and TAC). Although this targeted approach does not provide a comprehensive characterization of the phytochemical composition, it enables reliable comparison of representative phenolic markers among extraction systems, while additional non-quantified compounds may also contribute to the biological activities observed. The results indicate that vinegar-based systems, particularly under unbuffered acidic conditions and in combination with αCD, can influence the recovery and distribution of selected phenolic compounds extracted from pomegranate byproduct under the UAE conditions studied here. The stability of phenolic compounds during storage was not assessed in this study and should be considered in future work, particularly in relation to acidic extraction media.

#### 3.3.3. Antimicrobial Activity

Table 5 presents the antimicrobial activity results (inhibition zone diameter, mm) of pomegranate liquid extracts obtained after UAE using M50, unbuffered (V2) or buffered (sodium-based, VT1; potassium-based, VT2) distilled vinegar (0–10% αCD), against *Listeria monocytogenes* STCC 4032 and *Salmonella enterica* STCC 443. The inhibition observed for *L. monocytogenes* in the pomegranate methanolic extract (M50) (18.5 ± 0.9 mm) was associated with its relatively high TPC (182.0 ± 10.4 mg GAE/g DW) (Figure 7). UAE appeared to enhance the antimicrobial activity of the M50 extract, since the extraction solvent (methanol–water mixture without polyphenol enrichment) showed no detectable antimicrobial activity against either of the bacteria tested. In addition, the antimicrobial activity of M50 against *L. monocytogenes* (18.5 ± 0.9 mm) was higher than that obtained by conventional extraction (CE) (15.6 ± 0.5 mm) [24], in agreement with previous studies reporting improved bioactivity of sonication-assisted extracts [79]. It should be noted that the methanolic extracts showed significantly lower antimicrobial activity than the antibiotic used as a positive control (25.6 ± 0.3 and 20.2 ± 0.3 mm against *L. monocytogenes* and *S. enterica*, respectively).

Unbuffered distilled vinegar (V2) produced highly active extracts, with inhibition zones > 15 mm (7–10 mm, low; 11–14 mm, medium; and >15 mm, high antimicrobial activity) [80] against both *L. monocytogenes* (Gram-positive bacterium) (35.4 ± 1.0 mm) and *S. enterica* (Gram-negative bacterium) (23.1 ± 1.6 mm). The higher sensitivity observed in the Gram-positive bacterium is consistent with its simpler cell wall structure compared to the Gram-negative bacterium [81]. The antimicrobial activity of vinegar is well documented and is mainly attributed to acetic acid, often in combination with minor bioactive compounds in fruit vinegars (e.g., wine or cider vinegars), leading to broad antimicrobial effects against bacteria such as *Salmonella* spp., *Escherichia coli*, *Streptococcus pyogenes*, *Staphylococcus* spp., *Pseudomonas* spp., and *Klebsiella pneumoniae*, as well as fungi such as *Aspergillus* spp. [80,82]. In the present study, although the vinegar used was derived from alcohol fermentation and therefore lacked intrinsic phenolic compounds, its relatively high acetic acid content (9.6%, higher than the typical commercial values of 3–5% [65]) combined with polyphenol enrichment from the pomegranate byproduct after UAE likely contributed to the observed antimicrobial effects.

Overall, UAE combined with αCD incorporation (V2-10) resulted in an increased inhibitory effect, which may be associated with higher polyphenol availability in these extracts. However, no differences (*p* > 0.05) were observed between 10 and 15% αCD addition, consistent with the lack of further improvement in phenolic extraction (Figure 5). These results suggest a relationship between the chemical composition of the extractants and the antimicrobial properties of the resulting extracts [63], although a direct causal relationship cannot be fully established from the present data. The antimicrobial activity reported herein should be interpreted within the scope of the agar well-diffusion assay, which was employed as a comparative screening method for evaluating the different extracts.

The antimicrobial mechanisms of polyphenols are well described and include disruption of cell membranes, interaction with nucleic acids, and interference with microbial metabolism and virulence-related processes (e.g., biofilm formation and toxin production) [64,83,84,85,86]. In addition, interactions between polyphenols and sulfhydryl groups of bacterial proteins may contribute to enzyme inhibition and metabolic impairment, which could be consistent with the higher activity observed in extracts with greater TPC (V2 and V2-10) [64,84]. Previous studies have also reported inhibitory effects of anthocyanin-rich berry extracts against Gram-positive bacteria activity such as *L. monocytogenes* or *Staphylococcus aureus*, as well as Gram-negative bacteria, including *E. coli* and *S. enterica* ser. *Typhimurium* [84]. Catechins from green tea have also been reported to disrupt lipid bilayers and bacterial membrane integrity in *S. aureus* [84].

The inhibition zones obtained for *L. monocytogenes* with V2 and V2-10 extracts were higher (*p* < 0.05) than those observed for the methanolic and antibiotic controls. V2 extracts also showed significantly higher inhibition against *S. enterica* than controls. These findings suggest that both acetic acid and polyphenolic compounds may contribute to the overall antimicrobial activity. However, based on the present data, their interaction should be considered additive rather than synergistic in a strict statistical sense [67,80]. When acetic acid penetrates bacterial cells, it can dissociate in the cytoplasm, leading to intracellular acidification and disruption of metabolic processes, which ultimately results in bacterial growth inhibition [80]. This mechanism likely explains the strong antimicrobial activity observed in V2-based extracts.

Buffered pomegranate extracts inhibited the growth of *L. monocytogenes* (12.3–18.1 mm, medium-to-high antimicrobial activity). No differences (*p* > 0.05) were observed between these buffered extracts (12.3–18.1 mm) and M50 (18.5 ± 0.9 mm), although all remained significantly lower than the antibiotic control (25.5 ± 0.3 mm). Notably, while buffered vinegars (VT1 and VT2) without phenolic enrichment showed no antimicrobial activity, their corresponding pomegranate extracts exhibited measurable inhibition against the Gram-positive bacterium. In contrast, no activity was observed against *S. enterica*, likely due to the higher intrinsic resistance of Gram-negative bacteria associated with their outer membrane structure [81].

Acidity appears to play an important role in antimicrobial efficacy. Buffered vinegars with low acetic acid content (0.3–0.7%) and near-neutral pH (>6) showed reduced antimicrobial performance compared to V2 and V2-10 extracts. These differences, together with lower extraction efficiency under buffered conditions, may explain the reduced antimicrobial activity observed [63,67,80].

Although the type of salt (sodium, VT1; potassium, VT2) affected TPC (*p* < 0.05), it did not significantly influence antimicrobial activity when αCD was added (*p* > 0.05). The incorporation of αCD increased the antimicrobial activity of VT1-10 compared to VT1; however, no differences were detected among VT1-10, VT2, and VT2-10. Similar inhibitory effects of sodium-buffered vinegar have been reported in meat products such as deli-style turkey and chicken breast against *L. monocytogenes* [32,87].

Overall, the antimicrobial activity observed in this study appears to result from the combined contribution of acetic acid content and polyphenol concentration. Nevertheless, the relative contribution of each factor and its possible interaction should be interpreted cautiously. The buffering conditions likely influenced both the extraction efficiency and the chemical profile of the resulting extracts, thereby affecting their in vitro antimicrobial performance [63].

## 4. Conclusions

In this study, ultrasound-assisted extraction (UAE) combined with vinegar-based solvent systems and α-cyclodextrin (αCD) was evaluated as a food-compatible green strategy for the recovery of phenolic compounds from pomegranate peel byproduct. Compared with the conventional methanolic extraction used as a reference, the optimized vinegar-based systems demonstrated that representative phenolic compounds can be effectively recovered under UAE conditions while avoiding the use of organic solvents. The selected UAE conditions identified in this study were 15 min extraction at 30 °C using 10% αCD in combination with either V2 unbuffered vinegar or VT2 buffered vinegar. Among the evaluated systems, the non-buffered vinegar solvent (V2-10) showed the highest TPC and the highest concentrations of the selected phenolic markers. Targeted HPLC-QTOF-MS analysis confirmed punicalagin as the predominant quantified phenolic compound in all extracts and demonstrated that solvent composition and acidity were the main factors influencing phenolic recovery. The effect of αCD was solvent-dependent, with the greatest effects observed in buffered vinegar systems, whereas its influence under unbuffered acidic conditions was comparatively limited. Overall, the findings support the potential of vinegar-based solvent systems as sustainable food-compatible alternatives for the selective recovery of phenolic compounds from pomegranate peel byproduct. The proposed extraction strategy may contribute to the valorization of this agro-industrial byproduct through the development of phenolic-rich extracts suitable for potential food-related applications while reducing reliance on conventional organic solvents.

## Figures and Tables

**Table 1:** Evaluation of the experimental design of ultrasound-assisted extraction (UAE) from pomegranate peel powder (1:50 (*w*:*v*) powder:solvent ratio) under different parameters (temperature and time) using different solvents (W, ultrapure water; M50, methanol–ultrapure water (1:1; *v*:*v*); V1, vinegar with high (≈20%) acetic acid content; V2, vinegar with a moderate (≈10%) acetic acid content).

Parameter *	Solvent	Plant Byproduct Powder:Solvent Ratio (*w*:*v*)	UAE Temperature	UAE Time
Time	W, M50, V1, V2	1:50	30 °C	15, 30, 60 min
Temperature	W, M50, V1, V2	1:50	20, 30, 60 °C	15 min

* UAE experiments were conducted in two independent sets: (i) extraction time evaluation (15, 30, and 60 min at 30 °C), and (ii) extraction temperature evaluation (20, 30, and 60 °C at 15 min). Solid-to-solvent ratio was kept constant at 1:50 (*w*:*v*) in all experiments.

**Table 2:** Evaluation of the experimental design of ultrasound-assisted extraction (UAE; 30 °C, 15 min, 100% power) of pomegranate peel powder (1:50 (*w*:*v*) powder:solvent ratio) with different solvents (W, ultrapure water; M50, methanol–ultrapure water (1:1; *v*:*v*); M100, 100% methanol; V1, vinegar with high (≈20%) acetic acid content; V2, vinegar with a moderate (≈10%) acetic acid content) supplemented with different αCD concentrations (10–15%).

Nomenclature	αCD Content (%)	Solvent
W	-	Ultrapure water
W-10	10%	Ultrapure water
W-15	15%	Ultrapure water
M100	-	100% methanol
M50	-	50% methanol
V1	-	Unbuffered vinegar (20% acetic acid)
v1-10	10%	Unbuffered vinegar (20% acetic acid)
V1-15	15%	Unbuffered vinegar (20% acetic acid)
V2	-	Unbuffered vinegar (10% acetic acid)
V2-10	10%	Unbuffered vinegar (10% acetic acid)
V2-15	15%	Unbuffered vinegar (10% acetic acid)

**Table 3:** Evaluation of the experimental design of ultrasound-assisted extraction (UAE; 30 °C, 15 min, 100% power) of pomegranate powdered byproduct (1:50 (*w*:*v*) powder:solvent ratio) with different vinegars (unbuffered (V2) or buffered (sodium (VT1) or potassium salts (VT2)), compared to other solvents (M50, methanol–ultrapure water (1:1; *v*:*v*)), and supplemented with 10% αCD.

Nomenclature	αCD Content (%)	Solvent
M50	-	50% methanol
V2	-	Unbuffered vinegar (10% acetic acid)
V2-10	10%	Unbuffered vinegar (10% acetic acid)
VT1	-	Buffered vinegar (sodium salts)
VT1-10	10%	Buffered vinegar (sodium salts)
VT2	-	Buffered vinegar (potassium salts)
VT2-10	10%	Buffered vinegar (potassium salts)

**Table 4:** Quantification of selected phenolic compounds in pomegranate byproduct extracts (mg/g DW) obtained after UAE (30 °C, 15 min, 100% power) (mean (*n* = 3) ± standard deviation). M50 = methanol–ultrapure water (1:1); V2 = distilled vinegar with a moderate acetic acid content; V2-10 = V2 + 10% αCD; VT1 = sodium-buffered distilled vinegar; VT1-10 = VT1 + 10% αCD; VT2 = potassium-buffered distilled vinegar; VT2-10 = VT2 + 10% αCD.

Solvent	Gallic Acid	Ellagic Acid	Catechin	Punicalagin	Sum of Quantified Phenolic Markers
M50	6.3 ± 0.8 ^a^	1.9 ± 0.5 ^ab^	2.7 ± 0.2 ^a^	51.7 ± 10.1 ^ac^	62.5 ± 10.7 ^ac^
V2	5.8 ± 0.4 ^a^	1.9 ± 0.4 ^ab^	2.6 ± 0.1 ^a^	61.3 ± 5.5 ^ab^	71.6 ± 6.3 ^ab^
V2-10	6.3 ± 0.5 ^a^	2.3 ± 0.4 ^a^	3.0 ± 0.3 ^a^	64.5 ± 1.8 ^b^	76.2 ± 2.2 ^b^
VT1	3.4 ± 0.1 ^bd^	0.4 ± 0.1 ^c^	0.7 ± 0.1 ^b^	51.4 ± 3.0 ^ac^	55.9 ± 2.0 ^cd^
VT1-10	3.8 ± 0.1 ^b^	0.8 ± 0.1 ^cb^	1.6 ± 0.1 ^c^	44.0 ± 0.5 ^c^	50.2 ± 0.4 ^cd^
VT2	2.0 ± 0.2 ^c^	0.4 ± 0.1 ^c^	0.7 ± 0.1 ^b^	26.1 ± 1.6 ^d^	29.2 ± 1.3 ^e^
VT2-10	2.4 ± 0.1 ^cd^	1.3 ± 0.1 ^b^	1.7 ± 0.1 ^c^	41.7 ± 2.0 ^c^	47.1 ± 1.7 ^d^

Different letters in the same column indicate significant differences (*p* < 0.05) among treatments, according to one-way ANOVA followed by Tukey’s HSD test. The sum corresponds to the quantified phenolic markers included in this study and does not represent the total phenolic content of the samples.

**Table 5:** Inhibition zone diameter (mm) for pomegranate byproduct liquid extracts (1:50, weight:volume) after UAE (30 °C, 15 min, 100% power), against *Listeria monocytogenes* STCC 4032 and *Salmonella enterica* STCC 443 (mean (*n* = 3) ± standard deviation). M50 = methanol–ultrapure water (1:1); V2 = distilled vinegar with a moderate acetic acid content; V2-10 = V2 + 10% αCD; VT1 = sodium-buffered distilled vinegar; VT1-10 = VT1 + 10% αCD; VT2 = potassium-buffered distilled vinegar; VT2-10 = VT2 + 10% αCD.

Raw Material	Extraction Solvent	Inhibition Zone Diameter (mm)	
		*L. monocytogenes* STCC 4032	*S. enterica* STCC 443
Pomegranate	M50	18.5 ± 0.9 ^a^	NA ^a^
	V2	35.4 ± 1.0 ^b^	23.1 ± 1.6 ^b^
	V2-10	39.5 ± 0.4 ^c^	20.8 ± 1.0 ^c^
	VT1	12.3 ± 0.4 ^d^	NA ^a^
	VT1-10	17.1 ± 0.9 ^a^	NA ^a^
	VT2	18.0 ± 1.0 ^a^	NA ^a^
	VT2-10	18.1 ± 1.4 ^a^	NA ^a^
Oxytetracycline dihydrate, 100 mg/L	-	25.3 ± 0.6 ^d^	20.2 ± 0.3 ^c^

Different letters in the same column indicate significant differences (*p* < 0.05) among treatments, according to one-way ANOVA followed by Tukey’s HSD test. NA = no measurable activity observed. Oxytetracycline dihydrate (100 mg/L) was used as positive control. Negative control (sterilized distilled water) did not show any activity.

## Data Availability

The original contributions presented in the study are included in the article. Further inquiries can be directed to the corresponding author.
